# Synthesis of Tricyclic Condensed Rings Incorporating the Pyrazole or Isoxazole Moieties Bonded to a 4-Piperidinyl Substituent

**DOI:** 10.3390/molecules18078147

**Published:** 2013-07-10

**Authors:** Giansalvo Pinna, Giovanni Loriga, Gérard A. Pinna, Giorgio Chelucci

**Affiliations:** 1 Dipartimento di Chimica e Farmacia, Università di Sassari, Via F. Muroni 23/A, 07100 Sassari, Italy; E-Mail: pinger@uniss.it; 2 C.N.R. Istituto di Farmacologia Traslazionale, UOS Cagliari, Edificio 5, Loc Piscinamanna, 09010 Pula (CA), Italy; E-Mail: giovanni.loriga@ift.cnr.it; 3 Dipartimento di Agraria, Università di Sassari, Viale Italia 39, 07100 Sassari, Italy; E-Mail: chelucci@uniss.it

**Keywords:** heterocyclization, hydrazine, hydroxylamine, tricyclic isoxazoles, tricyclic pyrazoles

## Abstract

In this paper we report the synthesis of new compounds based on the pyrazole and isoxazole framework fused to a cycloalkene unit, and bearing as a substituent the 1-piperidinyl group as new examples of potential antipsychotic molecules. The general synthesis involves the acylation of a chloro-substituted cyclic ketone with a 1-substituted piperidine-4-carboxylate derivative, followed by heterocyclization of the formed 1,3-dioxo compound with a hydrazine or hydroxylamine.

## 1. Introduction

**Figure 1:**
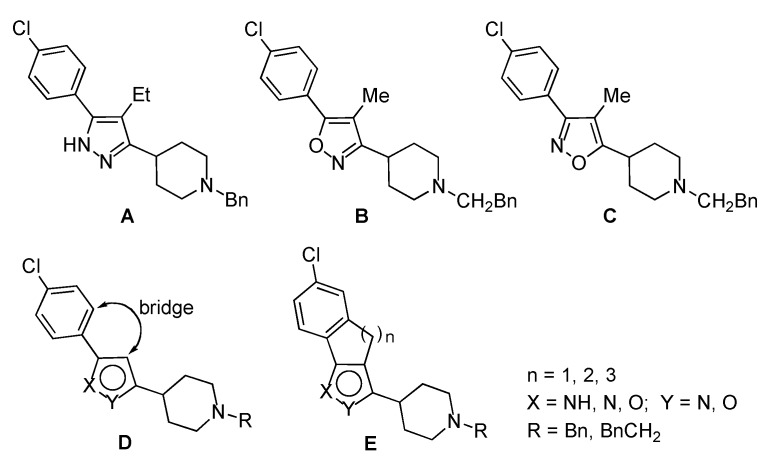
Leads and target molecules.

Among the compounds with antipsychotic properties [1] there are the heteropentalenes **A**–**C** [2], characterized by a pyrazole and isoxazole framework bonded to *p*-chlorophenyl and 4-piperidinyl substituents (Figure 1). In continuation of our interest in the field of the synthesis of biologically active compounds [3], we have now devoted our attention to obtain tricyclic compounds **E** related to the heteropentalenes **A**–**C** as new potential antipsychotic compounds. 

A well known strategy to affect the biological activity of organic compounds is to decrease their conformational flexibility. In fact, it has been proposed that appropriate structural constraints could restrict a pharmacophoric structural element to a sufficiently small region of conformational space thereby permitting the ligand to bind to its designated receptor with high affinity and selectivity [4,5]. A way to achieve this goal with heteropentalenes **A**–**C** could be to connect the unsubstituted central carbon of the heteropentalene and the α-carbon of the phenyl group with an alkylidene bridge (formula **D**, Figure 1).

In this line, we have developed a practical and extensible method to build compounds with a tricyclic framework incorporating the pyrazole and isoxazole framework and with the central ring that can be modulated in size, namely compounds with the general formula **E** shown in Figure 1. These new compounds share with **A**–**C** the chlorine on the aryl ring and the 4-(1-benzyl)- or 4-(1-phenylethyl)-piperidinyl substituents on the isoxazole and pyrazole moieties (Figure 1).

**Scheme 1:**
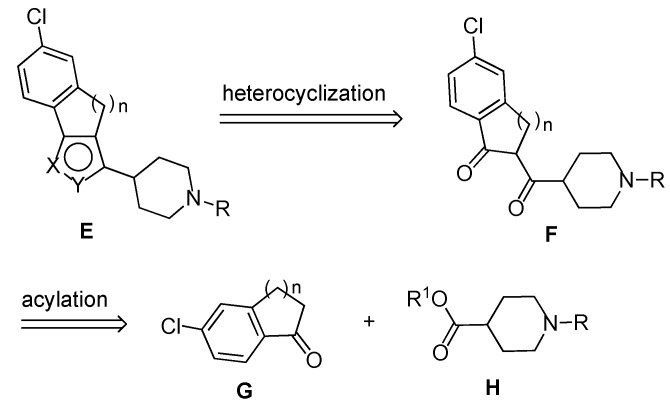
Retrosynthesis approach.

The planned retrosynthesis of the derivatives **E** is shown in Scheme 1. In this approach, the final heterocyclization of the 1,3-dioxo compounds **F** with hydrazine or hydroxylamine is preceded by acylation of the chloro-substituted cyclic ketones **G** with the 1-substituted piperidine-4-carboxylate derivatives **H**.

## 2. Results and Discussion

**Scheme 2:**
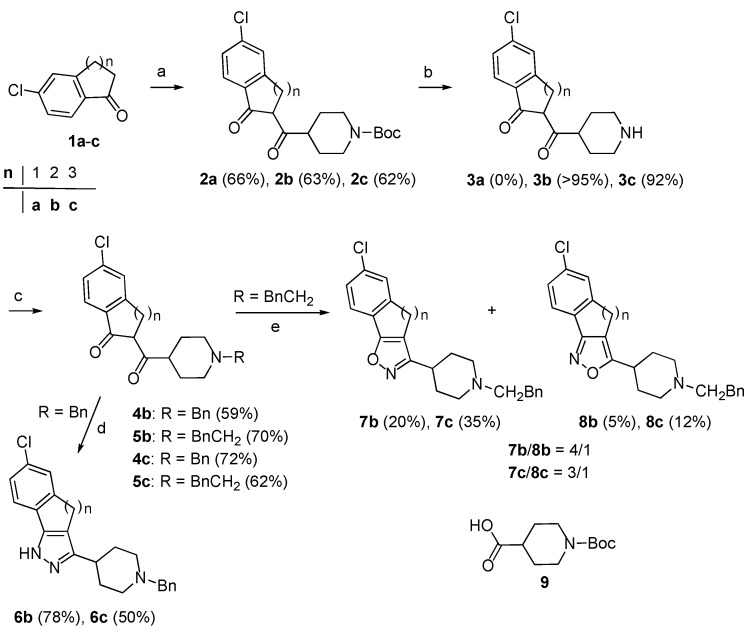
Synthesis of compounds **6b**, **6c**, **7b**, **7c**, **8b **and **8c**.

To begin the synthesis of the target derivatives **E** (Scheme 1), the known cyclic ketones **1a**–**c** [6,7] were acylated by reaction of the corresponding sodium enolate, obtained by reaction with sodium hydride, with the reagent formed by reaction of the *N*-Boc protected isonipecotic acid **9** with 1,1′-carbonyldiimidazole [8] (Scheme 2). In this way, 1,3-dicarbonyl derivatives **2a**–**c** were obtained in 62–63% yields. Next, these compounds were submitted to *N*-deprotection by treatment with trifluoroacetic acid in CH_2_Cl_2_. However, while **2b** and **2c** were easily deprotected giving compounds **3b** and **3c **in high yields (92–95%), the removal of the *N*-Boc group from **2a** failed. Further attempts to deprotect **2a** with HCOOH, 3N HCl in AcOEt, CF_3_COOH and Et_3_SiH, and SnCl_4_ in AcOEt all failed unexpectedly, therefore, alternative approaches to the target compounds **6a**, **7a** and **8a** were investigated next (see below).

Compounds **3b**,**c** were converted in 59–72% yields into the related *N*-benzyl and *N*-phenylethyl derivatives** 4b**,**c** and **5b**,**c** by reaction with benzyl chloride and 2-phenyl-1-iodoethane, respectively. With the key 1,3-dicarbonyl derivatives **4b**,**c** and **5b**,**c** in hand, their conversion into the desired derivatives **E** was pursued according to the planned retrosynthetic scheme. Compounds **4b**,**c** and hydrazine in methanol were stirred at room temperature to afford the pyrazole derivatives **6b** and **6c **in good yields (78% and 50%, respectively). Treatment of **5b**,**c** with hydroxylamine hydrochloride in EtOH/AcOH at 80 °C gave isoxazoles **7b**,**c** and **8b**,**c** as mixtures of regioisomers in moderate to good yields. With **5b **isoxazoles **7b** and **8b** were obtained in a 4/1 ratio, while **5c** gave isoxazoles **7c** and **8c **in a 3.2/1 ratio [9]. 

*Reagents and conditions*: (a) (i) NaH, DMF, rt; (ii) **9**, 1,1'-carbonyldiimidazole, DMF, rt, 45 min; (iii) 120 °C, 6 h; (b) CF_3_COOH, CH_2_Cl_2_, rt, 2 h; (c) BnCl or BnCH_2_I. DMF, *i*-Pr_2_NEt, 25–60 °C, 12h; (d) H_2_N-NH_2_, MeOH, rt, 12 h; (e) NH_2_OH, EtOH, AcOH, 80 °C, 12 h.

**Scheme 3:**
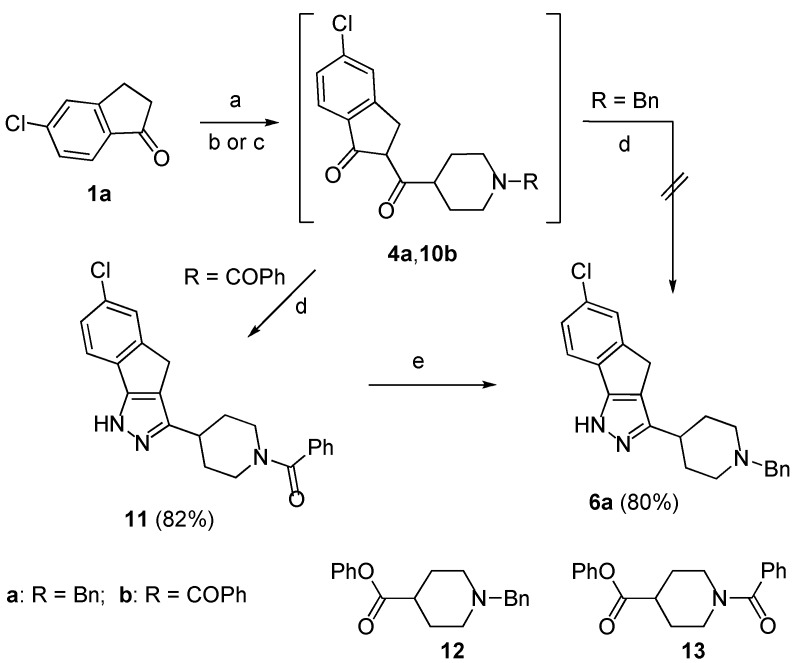
Synthesis of compound **6a**.

To obtain compound **6a** the synthetic routes outlined in Scheme 3 were followed. Firstly, the sodium enolate of the ketone **1a** was reacted with phenyl 1-benzylpiperidine-4-carboxylate **12**, but the 1,3-dicarbonyl intermediate **4a** failed to give the expected pyrazole **6a** by treatment with hydrazine in AcOH/MeOH at 80 °C. However, when the same enolate was treated with phenyl 1-(phenylcarbonyl)-piperidine-4-carboxylate **13** [10], obtained by esterification with phenol of the parent acid (Scheme 5), the formed 1,3-dicarbonyl **10b** afforded by treatment with hydrazine in AcOH/MeOH at 80 °C the substituted pyrazole **11** in 82% yield. Finally, LiAlH_4_ reduction of the carbonyl group to the methylene unit afforded the target pyrazole **6a** in 80% yield (66% overall yield from **1a**).

**Scheme 4:**
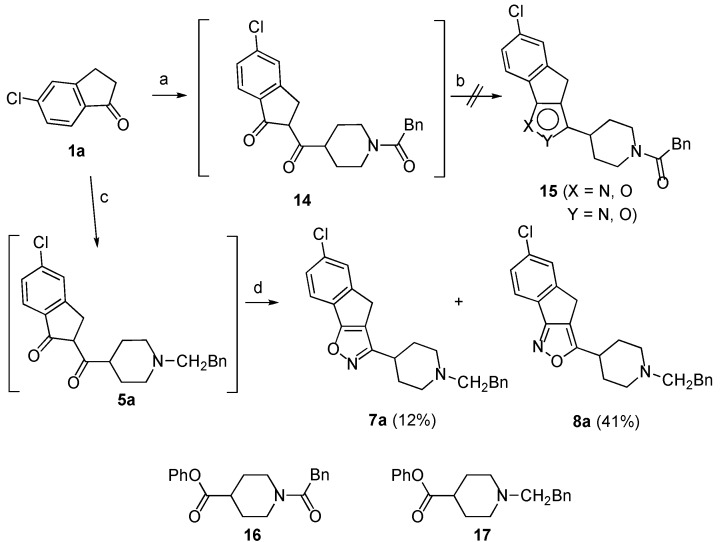
Synthesis of compounds **7a** and **8a**.

**Scheme 5:**
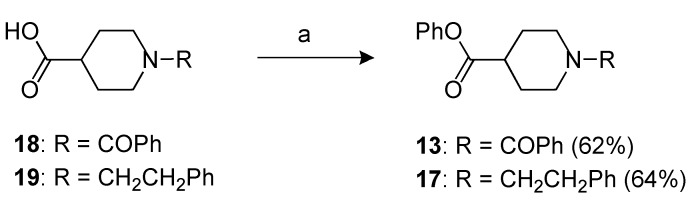
Synthesis of compounds **13** and **17**.

This satisfactory result appeared to open a way to isoxazoles **7a** and **8a** by simple replacing of the piperidine derivative **13** with the analogue **16** (Scheme 4). However, the treatment of the 1,3-dicarbonyl intermediate **14**, obtained in turn by reaction of **1a** with **16**, with hydroxylamine hydrochloride in EtOH/AcOH at 80 °C failed to afford the expected isoxazoles **15**. This unexpected result prompted us to verify another route based on the on the use of the *N*-benzylpyperidine **17** that was obtained by esterification with phenol of the parent acid (Scheme 5). We were pleased to find that the1,3-dicarbonyl intermediate **5a**, formed by reaction of the enolate of the ketone **1a** with **17**, could be directly converted in the usual way into a mixture of isoxazoles **7a** and **8a** in 41% and 12% yield, respectively (Scheme 4).

*Reagents and conditions*: (a) (i) NaH, benzene, rt, (b) **12**, reflux, 3.5 h; (c) **13**, reflux, 3.5 h; (d) H_2_N-NH_2_, AcOH, EtOH, 80 °C, 4 h; (e) LiAlH_4_, THF, rt, 12 h.

*Reagents and conditions*: (a) (i) NaH, benzene, rt, (ii) **16**, reflux; (b) NH_2_OH, AcOH, EtOH, 80 °C; (c) (i) NaH, benzene, rt, (ii) **17**, reflux, 4 h; (b) NH_2_OH, AcOH, EtOH, 80 °C, 7 h.

*Reagents and conditions*: (a) PhOH, EDC, DMAP, CH_2_Cl_2_, 40 °C, 14 h.

## 3. Experimental

### 3.1. General

All reagents and solvents were purchased from commercial suppliers and used as received. Low boiling petroleum ether corresponds to the fraction collected between 40 and 60 °C. THF was distilled from sodium-benzophenone ketyl and degassed thoroughly with dry nitrogen directly before use. Melting points were determined on a Büchi 510 capillary apparatus and are uncorrected. IR spectra were recorded on a J ASCO FT/IR-460 plus equipment.The NMR spectra were obtained with a Varian VXR-300 spectrometer at 200 MHz for ^1^H and 50 MHz for ^13^C. Chemical shifts are reported in ppm downfield from internal Me_4_Si in CDCl_3_. The following abbreviations were used to describe peak patterns where appropriate: singlet (s), doublet (d), triplet (t), multiplet (m) and broad resonances (br). Elemental analyses were performed on a Perkin-Elmer 240 B analyser. TLC was performed on Merck silica gel 60 TLC plates F254 and visualized using UV or phosphomolibdic acid. Flash chromatography was carried out on silica gel (40–60 mesh). The chloroketone **1a** was a commercial compound. 6-Chloro-3,4-dihydronaphthalen-1-one (**1b**) [6], 7-chloro-2,3,4,5-tetrahydrobenzocyclo-heptan-1-one (**1c**) [7], *N*-Boc-nipecotic acid [8] and the piperidines **18** [10] and **19** [11] were obtained following the corresponding literature procedures. 

### 3.2. General Procedure for the Synthesis of the Compounds **2a–2c**

A solution of 1-(*tert*-butoxycarbonyl)piperidine-4-carboxylic acid (**9**, 2.85 g, 12.45 mmol) and 1,1′-carbonyldiimidazole (2.29 g, 14.11 mmol) in DMF (3 mL) was stirred at room temperature for 45 min. This solution was added dropwise to a solution prepared by stirring for 20 min the suitable ketone **1a**, **1b** or **1c** (7.64 mmol) with NaH (60% in oil, 0.93 g, 23.20 mmol) in DMF (20 mL). The resulting mixture was heated for the appropriate time. After cooling, H_2_O was added and the mixture was extracted with Et_2_O (3 × 30 mL). The organic phase was dried over Na_2_SO_4_, filtered and the solvent was removed under reduced pressure. The residue was purified by flash chromatography.

*tert-Butyl 4-(5-chloro-1-oxo-2,3-dihydro-1H-indene-2-carbonyl)piperidine-1-carboxylate* (**2a**). According to the general procedure, the reaction between **1a** and **9** was carried out at 30 °C for 7 h. The residue was purified by flash chromatography (petroleum ether/EtOAc = 9:1) affording **2a**: yield 66%; red solid; Mp 102–103 °C. *R_f_ =* 0.10 (petroleum ether/AcOEt = 9:1). ^1^H-NMR: δ 1.48 (s, 9H), 1.60–1.95 (m, 4H), 2.40–2.64 (m, 1H), 2.64–2.98 (m, 2H), 3.62 (s, 2H), 4.13–4.33 (m, 2H), 7.40 (d, 1H, *J* = 8.0 Hz), 7.48 (s, 1H), 7.74 (d, 1H, *J* = 8.0 Hz), 13.80 (brs, 1H). ^13^C-NMR: δ 27.9 (CH_2_), 28.4 (3 × CH_3_), 29.8 (2 × CH_2_), 41.8 (CH), 43.4 (2 × CH_2_), 79.7 (C), 108.8 (C), 124.2 (CH), 126.0 (CH), 128.1 (CH), 136.7 (C), 139.1 (C), 148.6 (C), 154.7(CO), 182.7 (CO), 191.3 (COH). IR: (nujol) ν 1703 (CO), 1655 (CO), 1605 (CO) cm^−^^1^. Anal. Calcd for C_20_H_24_ClNO_4_: C, 63.57; H, 6.40; N, 3.71. Found: C, 63.65; H, 6.49; N, 3.81.

*tert-Butyl 4-(6-chloro-1-oxo-1,2,3,4-tetrahydronaphthalene-2-carbonyl)piperidine-1-carboxylate* (**2b**). According to the general procedure, the reaction between **1b** and **9** was carried out at 110 °C for 7 h. The residue was purified by flash chromatography (petroleum ether/EtOAc = 8:2) affording **2b**: yield 63%; red solid; Mp 120–121 °C. *R_f_ =* 0.43 (petroleum ether/AcOEt = 8:2). ^1^H-NMR: δ 1.47 (s, 9H), 1.59–1.87 (m, 5H), 2,67 (d, 2H, *J* = 7.4 Hz), 2.80–2.88 (m, 4H), 4.18 (d, 2H, *J* = 7.4 Hz), 7.24 (d, 1H, *J* = 10.2 Hz), 7.31 (s, 1H), 7.87 (d, 1H, *J* = 8.2 Hz), 16.65 (s, 1H). ^13^C-NMR: δ 21.9 (CH_2_), 27.8 (CH_2_), 28.4 (3 × CH_3_), 29.3 (2 × CH_2_), 41.4 (CH), 43.3 (2 × CH_2_), 79.6 (C), 104.6 (C), 126.2 (CH), 127.2 (CH), 127.3 (CH), 127.5 (C), 136.7 (C), 142.2 (C), 157.3 (CO), 179.0 (CO), 197.8 (COH). IR: (nujol) ν 1707 (CO), 1650 (CO), 1611 (CO) cm^−^^1^. Anal. Calcd for C_21_H_26_ClNO_4_: C, 64.36; H, 6.69; N, 3.57. Found: C, 64.88; H, 6.65; N, 3.59.

*tert-Butyl 4-(2-chloro-5-oxo-6,7,8,9-tetrahydro-5H-benzo[7]annulene-6-carbonyl)piperidine-1-carboxylate* (**2c**). According to the general procedure the reaction between **1c** and **9** was carried out at 70 °C for 7 h. The residue was purified by flash chromatography (petroleum ether/EtOAc = 9:1) affording **2c**: yield 63%; yellow solid; Mp 134–136 °C. *R_f_ =* 0.31 (petroleum ether/AcOEt = 9:1). ^1^H-NMR: δ 1.48 (s, 9H), 1.53–1.92 (m, 7H), 1.92–2.13 (m, 1H), 2.18 (t, 2H, *J* = 6.8 Hz), 2.55–2.90 (m, 3H), 4.10–4.31 (m, 2H), 7.21 (s, 1H), 7.27–7.42 (m, 1H), 7.57 (d, 1H, *J* = 8.2 Hz), 16.78 (s, 1H). ^13^C-NMR: δ 22.7 (CH_2_), 28.3 (3 × CH_2_), 28.5 (CH_3_), 31.1 (CH_2_), 31.3 (2 × CH_2_), 40.9 (CH), 43.2 (2 × CH_2_), 79.5 (C), 108.2 (C), 126.8 (CH), 128.7 (CH), 129.0 (CH), 131.0 (C), 136.4 (C), 141.4 (C), 154.5 (CO), 186.2 (CO), 194.4 (COH). IR: (nujol) ν 1706 (CO), 1652 (CO), 1613 (CO) cm^−^^1^. Anal. Calcd for C_22_H_28_ClNO_4_: C, 65.10; H, 6.95; N, 3.45. Found: C, 66.08; H, 6.98; N, 3.42.

### 3.3. General Procedure for the Synthesis of Compounds **3b**, **3c**

A solution of CF_3_COOH (1.46 g, 12.8 mmol) in CH_2_Cl_2_ (4.6 mL) was added dropwise to a solution of the 1,3-dicarbonyl compound **2b** or **2c** (1.28 mmol) in CH_2_Cl_2_ (9.2 mL). After stirring 2 h at room temperature, CH_2_Cl_2_ was added. The resulting mixture was washed two times with a 10% solution of K_2_CO_3_ and then with H_2_O. The organic phase was dried over Na_2_SO_4_, filtered and the solvent was removed under reduced pressure. The residue was purified by flash chromatography.

*6-Chloro-2-(piperidine-4-carbonyl)-3,4-dihydronaphthalen-1(2H)-one* (**3b**). Compound **2b** was converted into the title product **3b** according to the general procedure. The residue was purified by flash chromatography (CHCl_3_/MeOH = 8:2) affording **3b**: yield 63%; yellow solid; Mp 150–154 °C. *R_f_ =* 0,10 (CHCl_3_/MeOH 8:2); ^1^H-NMR: δ 2.62–2.85 (m, 4H), 2.58–2.77 (m, 4H), 2.80–2.95 (m, 4H), 3.10-3.34 (m, 2H), 7.21 (s, 1H), 7.32 (d, 1H, *J* = 8.2 Hz), 7.86 (d, 1H, *J* = 8.2 Hz), 8.52–9.20 (brs, 1H). ^13^C-NMR: δ 21.9 (CH_2_), 27.5 (CH_2_), 31.3 (2 × CH_2_), 42.0 (CH), 45.1 (2 × CH_2_), 109.2 (C), 126.3 (CH), 127.4 (CH), 129.1 (CH), 137.0 (C), 137.2 (C), 144.5(C), 189.0 (CO), 192.4 (COH) IR: (nujol) ν 3453 (NH), 1701 (CO), 1680 (CO) cm^−^^1^. Anal. Calcd for C_16_H_18_ClNO_2_: C, 65.86; H, 6.22; N, 4.80. Found: C, 65.56; H, 6.26; N, 4.83.

*2-Chloro-6-(piperidine-4-carbonyl)-6,7,8,9-tetrahydro-5H-benzo[7]annulen-5-one* (**3c**). Compound **2c** was converted into the title product **3c** according to the general procedure. The residue was purified by flash chromatography (CHCl_3_/MeOH = 8:2) affording **3c**: yield 63%; white solid; Mp 138–142 °C. *R_f_ =* 0,11 (CHCl_3_/MeOH = 8:2); ^1^H-NMR: δ 1.60–1.92 (m, 4H), 1.92–2.10 (m, 2H), 2.18 (t, 2H, *J* = 6.2 Hz), 2.58–2.85 (m, 5H), 2.90–2.98 (m, 1H), 3.12–3.28 (m, 2H), 7.21 (s, 1H), 7.33 (d, 1H, *J* = 8.0 Hz), 7.56 (d, 1H, *J* = 8.0 Hz), 8.00–9.00 (brs, 1H). ^13^C-NMR: δ 22.8 (CH_2_), 28.7 (CH_2_), 31.1 (2 × CH_2_), 31.5 (CH), 40.6 (CH_2_), 45.1 (2 × CH_2_), 108.2 (C), 126.8 (CH), 128.7 (CH), 129.0 (CH), 136.0 (C), 136.8 (C), 141.5 (C), 188.0 (CO), 194.4 (COH). IR: (nujol) ν 3,430 (NH), 1,703 (CO), 1,680 (CO) cm^−^^1^. Anal. Calcd for C_17_H_20_ClNO_2_: C, 66.77; H, 6.59; N, 4.58. Found: C, 67.37; H, 6.64; N, 4.53.

### 3.4. General Procedure for the Synthesis of the Compounds **4b**, **4c** and **5b** **5c**

To a solution of the 1,3-dicarbonyl compound **3b** or **3c** (3.27 mmol) in DMF (18.25 mL) was added *i*-Pr_2_NEt (0.59 g, 4.58 mmol) and then the appropriate halide (1.1 eq). The mixture was then stirred at room temperature or heated under reflux for the necessary time. Water was added and the mixture was extracted with AcOEt. The organic phase was washed with brine, dried over Na_2_SO_4_, filtered and the solvent was removed under reduced pressure. The residue was purified by flash chromatography.

*2-(1-Benzylpiperidine-4-carbonyl)-6-chloro-3,4-dihydronaphthalen-1(2H)-one* (**4b**). A solution of the ketone **3b** and benzyl chloride in DMF was stirred at room temperature for 12 h. After workup the residue was purified by flash chromatography (petroleum ether/EtOAc = 1:1) affording **4b**: yield 59%; brown oil; *R_f_* *=* 0.46 (petroleum ether/EtOAc = 1:1); ^1^H-NMR: δ 1.51–1.80 (m, 4H), 1.90 (d, 2H, *J* = 11 Hz), 2.03 (d, 2H, *J* = 13.2 Hz), 2.58–2.76 (m, 1H), 2.84 (t, 2H, *J* = 7.4 Hz), 3.00 (d, 2H, *J* = 9.6 Hz), 3.55 (s, 2H), 7.20 (s, 1H), 7.29–7.40 (m, 6H), 7.86 (d, 1H, *J* = 8.6 Hz), 16.68 (s, 1H). ^13^C-NMR: δ 22.8 (CH_2_) , 28.6 (CH_2_), 32.1 (2 × CH_2_), 33.5 (CH), 45.1 (2 × CH_2_), 64.5 (CH_2_), 118.4 (C), 126.7 (CH), 126.9 (CH), 127.5 (CH), 128.3 (2 × CH), 128.6 (CH), 128.9 (CH), 129.1 (C), 129.3 (CH), 131.2 (C), 139.6 (C), 142.3 (C), 184.9 (CO), 195.3 (COH) IR: (nujol) ν 1,710 (CO), 1,682 (CO) cm^−1^. Anal. Calcd for C_23_H_24_ClNO_2_: C, 72.34; H, 6.33; N, 3.67. Found: C, 72.41; H, 6.38; N, 3.75.

*6-(1-Benzylpiperidine-4-carbonyl)-2-chloro-6,7,8,9-tetrahydro-5H-benzo[7]annulen-5-one* (**4c**). A solution of the ketone **3c** and benzyl chloride in DMF was stirred at room temperature for 12 h. After workup, the residue was purified by flash chromatography (petroleum ether/EtOAc = 1:1) affording **4c**: yield 72%; brown oil; *R_f_ =* 0.37 (petroleum ether/EtOAc = 1:1); ^1^H-NMR: δ 1.61–2.24 (m, 8H), 2.53–2.76 (m, 2H), 2.76–3.11 (m, 5H), 3.54 (s, 2H), 7.00–7.48 (m, 6H), 7.55 (d, 1H, *J* = 8.2 Hz), 8.00 (s, 1H), 16.8 (s, 1H). ^13^C-NMR: δ 22.9 (CH_2_), 28.5 (CH_2_), 31.0 (2 × CH_2_), 31.4 (CH_2_), 31,6 (CH), 52.8 (2 × CH_2_), 62.9 (CH_2_), 108.4 (C), 126.6 (CH), 126.8 (CH), 127.2 (CH), 127.5 (CH), 128.0 (CH), 128.3 (2 × CH), 128.7 (CH), 129.3 (C), 131.0 (C), 139.6 (C), 145.1 (C), 194.9 (CO), 195.3 (COH). Anal. IR: (nujol) ν 1,705 (CO), 1,682 (CO) cm^−1^. Calcd for C_24_H_26_ClNO_2_: C, 72.81; H, 6.62; N, 3.54. Found: C, 72.21; H, 6.65; N, 3.57.

*6-Chloro-2-(1-phenethylpiperidine-4-carbonyl)-3,4-dihydronaphthalen-1(2H)-one* (**5b**). A solution of the ketone **3b** and phenylethyl iodide in DMF was heated at 60 °C for 12 h. After workup, the residue was purified by flash chromatography (petroleum ether/EtOAc = 2:8) affording **5b**: yield 70%; brown oil;*R_f_ =* 0.42 (petroleum ether/EtOAc = 1:1); ^1^H-NMR: δ 1.26–2.53 (m, 11H), 2.54–2.75 (m, 2H), 2.75–2.99 (m, 2H), 3.04–3.23 (m, 2H), 7.08–7.45 (m, 6H), 7.49 (m, 1H), 7.71 (d, 1H, *J* = 9.0 Hz), 14,27 (s, 1H). ^13^C-NMR: δ 22.8 (CH_2_), 28.7 (CH_2_), 32.1 (CH_2_), 32.6 (2 × CH_2_), 33.5 (CH), 45.3 (2 × CH_2_), 64.5 (CH_2_) , 117.9 (C), 126.6 (CH), 128.3 (CH), 128.8 (CH), 128.9 (2 × CH), 129.1 (2 × CH), 129.2 (CH), 131.2 (C), 139.6 (C), 141.5 (C), 142.3 (C), 194.9 (CO), 195.3 (COH) Anal. IR: (nujol) ν 1,700 (CO), 1,681 (CO) cm^−1^. Calcd for C_24_H_26_ClNO_2_: C, 72.81; H, 6.62; N, 3.54. Found: C, 72.11; H, 6.66; N, 3.58.

*2-Chloro-6-(1-phenethylpiperidine-4-carbonyl)-6,7,8,9-tetrahydro-5H-benzo[7]annulen-5-one* (**5c**). A solution of the ketone **3c** and phenylethyl iodide in DMF was heated at 60 °C for 12 h. After workup, the residue was purified by flash chromatography (CHCl_3_/acetone = 9:1) affording **5c**: yield 62%; brown oil; *R_f_ =* 0.33 (CHCl_3_/acetone = 9:1); ^1^H-NMR: δ 1.72–1.89 (m, 3H), 1.95–2.28 (m, 7H), 2.53–2.92 (m, 7H), 3.12 (d, 2H, *J* = 9.6), 7.20 (s, 1H), 7.23–7.25 (m, 6H), 7.56 (d, 1H, *J* = 8.4 Hz), 16.7 (s, 1H). ^13^C-NMR: δ 22.8 (CH_2_), 28.8 (CH_2_), 31.2 (CH_2_), 31.7 (2 × CH_2_), 33.5 (CH_2_), 41.0 (CH), 53.2 (2 × CH_2_), 60.7 (CH_2_), 108.3 (CH), 126.0 (CH), 126.6 (CH), 126.8 (CH), 127.5 (2 × CH), 128.3 (CH), 128.6 (2 × CH), 128.7 (C), 129.1 (C), 131.0 (C), 141.5 (C), 187.9 (CO), 195.3 (COH). IR: (nujol) ν 1,699 (CO), 1,676 (CO) cm^−1^. Anal. Calcd for C_25_H_28_ClNO_2_: C, 73.25; H, 6.88; N, 3.42. Found: C, 73.76; H, 6.84; N, 3.46.

### 3.5. General Procedure for the Synthesis of Compounds **6b**, **6c**

A solution of the 1,3-dicarbonyl compound **4b** or **4c** (0,68 mmol) and hydrazine hydrate (0.32 g, 6,39 mmol) in MeOH (9 mL) was stirred overnight at room temperature. Water was added and the mixture was extracted with ethyl acetate. The organic phase was dried over anhydrous Na_2_SO_4_, filtered and the solvent was removed under reduced pressure. The residue was purified by flash chromatography.

*3-(1-Benzylpiperidin-4-yl)-7-chloro-4,5-dihydro-1H-benzo[g]indazole* (**6b**). Compound **4b** was converted into the title product **6b** according to the general procedure. After workup, the residue was purified by flash chromatography (CHCl_3_/acetone = 9:1) affording **6b**: yield 78%; yellow solid; Mp 173–174 °C; *R_f_ =* 0.51 (CH_2_Cl_2_/MeOH = 95:5); ^1^H-NMR: δ 1.80–2.27 (m, 6H), 2.71–2.82 (m, 3H), 2.91 (t, 2H, *J* = 7.2 Hz), 3.01 (t, 2H, *J* = 7.2 Hz), 3.56 (s, 2H), 7.20–7.40 (m, 7H), 7.64–7.71 (m, 1H). ^13^C-NMR: δ 18.9 (CH_2_), 29.6 (CH_2_), 31.3 (2 × CH_2_), 33.7 (CH), 53.6 (2 × CH_2_), 63.3 (CH_2_), 111.2 (C) 123.2 (CH), 126.9 (CH), 127.1 (CH), 127.4 (CH), 127.5 (C), 128.2 (2 × CH), 129.2 (2 × CH), 132.7 (C), 137.8 (C), 138.3 (C). 142.3 (CN), 142.8 (CN). Anal. Calcd for C_23_H_24_ClN_3_: C, 73.10; H, 6.40; N, 11.12. Found: C, 73.91; H, 6.43; N, 11.07.

*3-(1-Benzylpiperidin-4-yl)-8-chloro-1,4,5,6-tetrahydrobenzo[3,4]cycloepta[2,1-c]pyrazole* (**6c**). Compound **4c** was converted into the title product **6c** according to the general procedure. After elaboration, the residue was purified by flash chromatography (CHCl_3_/acetone = 9:1) affording **6c**: yield 50%; yellow solid; Mp 165–166 °C; *R_f_ =* 0.51 (CH_2_Cl_2_/MeOH = 95:5); ^1^H-NMR: δ 1.68–2.32 (m, 8H), 2.51–2.90 (m, 5H), 3.04 (d, 2H, *J* = 9.8 Hz), 3.59 (s, 2H), 7.10–7.42 (m, 7H), 7.60–7.72 (m, 1H), 9.10–10,01 (brs, 1H). ^13^C-NMR: δ 24.1 (CH_2_), 26.9 (CH_2_), 29.7 (CH_2_), 31.0 (2 × CH_2_), 34.8 (CH), 53.8 (2 × CH_2_), 63.2 (CH_2_), 112.5 (C), 125.7 (CH), 126.4 (CH), 127.1 (CH), 127.4 (CH), 127.5 (2 × CH), 128.2 (2 × CH), 129.3 (2 × C), 129.6 (C), 134.5 (C) 141.3 (CN, 142.8 (CN). Anal. Calcd for C_24_H_26_ClN_3_: C, 73.55; H, 6.69; N, 10.72 Found: C, 73.25; H, 6.88; N, 10.42. 

### 3.6. 1H-1-Oxa-2-aza-7-chloro-3-(1-phenethylpiperidin-4-yl)-4,5-dihydronaphto[2,1-d]isoxazole **(7b)** and 1H-1-oxa-2-aza-8-chloro-3-(1-phenethylpiperidin-4-yl)-5,6-dihydro-4H-benzo[3,4]cycloepta[1,2-d]isoxazole **(7c)**

A solution of the 1,3-dicarbonyl compound **5b** or **5c** (2.44 mmol) and hydroxylamine hydrochloride (1.02 g, 14.64 mmol) in EtOH (12.2 mL) containing 4 drops of AcOH was heated under reflux for 24 h. Water was added and the mixture was extracted with CHCl_3_. The organic phase was dried over Na_2_SO_4_, filtered and the solvent was removed under reduced pressure. The residue was purified by flash chromatography (CHCl_3_/MeOH = 97:3) to give **7b** or **7c**.

*Compound* **7b**. Yield 20%; brown solid; Mp 170–172 °C; *R_f_ =* 0.11 (CHCl_3_/MeOH = 97:3); ^1^H-NMR (DMSO-d_6_): δ 1.50–1.80 (m, 9H), 1.42–2.10 (m, 4H), 2.48 (t, 2H, *J* = 7.0 Hz), 2.69 (t, 2H, *J* = 7.0 Hz), 7.12–7.42 (m, 8H). ^13^C-NMR: δ 28.4 (CH_2_), 29.5 (CH_2_), 32.7 (CH_2_), 38.2 (2 × CH_2_), 40.7 (CH), 52.7 (2 × CH_2_), 59.7 (CH_2_), 117.8 (C), 125.0 (CH), 125.4 (CH), 125.8 (CH), 126.5 (CH), 127.9 (2 × CH), 128.2 (2 × CH), 128.6 (C), 130.9 (C), 136.4 (C), 144.1 (C), 157.3 (CN), 166.1 (CO). Anal. Calcd for C_24_H_25_ClN_2_O: C, 73.36; H, 6.41; N, 7.13. Found: C, 73.96; H, 6.44; N, 7.10. 

*Compound* **7c**. yield 35%; brown solid; Mp 114–117 °C; *R_f_ =* 0.24 (CHCl_3_/MeOH = 8:2); ^1^H-NMR (DMSO-d_6_): δ 1.82–2.10 (m, 4H), 2.11–2.33 (m, 4H), 2.60–2.76 (m, 5H), 2.70–2.98 (m, 4H), 3.10–3.21 (m, 2H), 7.12–7.20 (m, 7H), 7.88 (d, 1H, *J* = 8.2 Hz). ^13^C–NMR: δ 23.9 (CH_2_), 24.5 (CH_2_), 29.8 (2 x CH_2_), 33.6 (CH_2_), 35.2 (CH), 53.5 (2 x CH_2_), 60.7 (CH_2_), 113.8 (C), 126.1 (CH), 126.7 (CH), 128.1 (CH), 128.2 (CH), 128.3 (2 x CH), 128.6 (2 x CH), 129.6 (C), 134.7 (C), 141.5 (C), 142.1 (C), 161.2 (CN), 166.6 (CO) Anal. Calcd for C_25_H_27_ClN_2_O: C, 73.79; H, 6.69; N, 6.88. Found: C, 73.25; H, 6.71; N, 6.91. 

### 3.7. 2H-1-Aza-2-oxa-7-chloro-3-(1-phenethylpiperidin-4-yl)-4,5-dihydronaphto[1,2-c]isoxazole **(8b)** and 2H-1-aza-2-oxa-8-chloro-3-(1-phenethylpiperidin-4-yl)-5,6-dihydro-4H-benzo[3,4]cycloepta[2,1-c]isoxazole **(8c)**

A solution of the 1,3-dicarbonyl compound **5b** or **5c** (2.44 mmol) and hydroxylamine hydrochloride (1.02 g, 14.64 mmol) in EtOH (12.2 mL) containing 15 drops of AcOH was heated under reflux for 24 h. After cooling, H_2_O was added and the mixture was extracted with CHCl_3_. The organic phase was dried over Na_2_SO_4_, filtered and the solvent was removed under reduced pressure. The residue was purified by flash chromatography (CHCl_3_/acetone = 8:2) to give **8b** or **8c**.

*Compound* **8b**. Yield 5%; brown solid; Mp 165–166 °C; *R_f_ =* 0.24 (CHCl_3_/acetone = 97:3); ^1^H-NMR (CDCl_3_/DMSO-d_6_): δ 1.43–1.70 (m, 4H), 1.71–1.93 (m, 4H), 1.95–2.10 (m, 1H), 2.42–2.65 (m, 4H), 2.52 (t, 2H, *J* = 7.2 Hz), 2.65 (t, 2H, *J* = 7.2 Hz), 7.05–7.38 (m, 5H), 7.42–7.84 (m, 2H), 8.32 (d, 1H, *J* = 9.2 Hz). ^13^C–NMR (CDCl_3_/DMSO-d_6_): δ 28.3 (CH_2_), 29.5 (CH_2_), 32.6 (CH_2_), 38.2 (2 × CH_2_), 40.7 (CH), 53.1 (2 × CH_2_), 59.8 (CH_2_), 118.0 (C), 120.2 (CH), 124.1 (CH), 125.4 (CH), 125.0 (CH), 125.8 (2 × CH), 127.9 (2 × CH), 128.6 (C), 131.2 (C), 134.8 (C), 140.3 (C), 161.2 (CN), 167.9 (CO). Anal. Calcd for C_24_H_25_ClN_2_O: C, 73.36; H, 6.41; N. 7.13. Found: C, 72.86; H, 6.44; N. 7.01. 

*Compound* **8c**. Yield 12%; light brown solid; Mp 125–126 °C; *R_f_ =* 0.47 (CHCl_3_/acetone = 8:2); ^1^H-NMR: δ 1.95–2.38 (m, 8H), 2.52–2.78 (m, 5H) 2.78–3.01 (m, 4H), 3.15 (d, 2H, *J* = 9.6 Hz), 7.08–7.40 (m, 7H), 7.89 (d, 1H, *J* = 8.4 Hz). ^13^C-NMR: δ 20.7 (CH_2_), 27.0 (CH_2_), 29.2 (CH_2_), 33.0 (2 × CH_2_), 33.2, (CH_2_), 39.3 (CH), 53.1 (2 × CH_2_), 60.4 (CH_2_), 111.2 (C), 126.3 (CH), 126.7 (CH), 126.8 (CH), 127.8 (CH), 128.5 (2 × CH), 128.7 (2 × CH_2_), 129.2 (C), 129.7 (C), 135.2 (C), 142.7 (C), 161.8 (CN), 170.4 (CO). Anal. Calcd for C_25_H_27_ClN_2_O: C, 73.79; H, 6.69; N, 6.88. Found: C, 73.19; H, 6.62; N, 6.83.

*(4-(6-Chloro-1,4-dihydroindeno[1,2-c]pyrazol-3-yl)piperidin-1-yl)(phenyl)metanone* (**11**). 5-Chloro-2,3-dihydro-1*H*-inden-1-one **1a** (0.2 g, 1.23 mmol) and NaH (60% in oil, 0.12 g, 3.08 mmol) were added in sequence to a solution of phenyl 1-(phenylcarbonyl)piperidine-4-carboxylate **13** (0.33 g, 1.23 mmol) and the resulting mixture was heated under reflux for 3.5 h. After cooling a 50% aqueous solution of acetic acid was added and the resulting mixture was concentrated under reduced pressure. The residue was taken up in EtOH (4 mL) and AcOH (0.21 mL, 3.69 mmol). Hydrazine hydrate (0.09 mL, 1.85 mmol) was added and the resulting mixture was heated under reflux for 4 h. After cooling, the solvent was evaporated under reduced pressure and the residue was taken up in CH_2_Cl_2_. The organic phase was dried over Na_2_SO_4_, filtered and the solvent removed under reduced pressure. The residue was purified by flash chromatography (CHCl_3_/acetone = 95:5) affording **11**: yield 82%; yellow solid; Mp 167–170 °C; *R_f_ =* 0,24 (CHCl_3_/MeOH = 95:5); ^1^H-NMR: δ 1.58–2.21 (m, 6H), 2.90–3.21 (m, 3H), 3.63 (s, 2H), 7.33 (d, 1H, *J* = 8.0 Hz), 7.37–7.50 (m, 6H), 7.6 (d, 1H, *J* = 8.0 Hz). ^13^C-NMR: δ 27.3 (CH_2_), 29.1 (2 × CH_2_), 29.4 (CH), 34.2 (2 × CH_2_), 120.7 (C), 126.2 (CH), 126.9 (CH), 127.3 (CH), 128.5 (2 × CH), 129.8 (2 × CH_2_), 129.9 (CH), 132.1(C), 132.7 (C), 133.8 (C), 141.6 (C), 150.1 (CN) 157.3 (CN), 170.5 (CO). Anal. Calcd for C_22_H_20_ClN_3_O: C, 69.93; H, 5.33; N, 11.12. Found: C, 70.34; H, 5.31; N, 11.17. 

*3-(1-Benzylpiperidin-4-yl)-6-chloro-1,4-dihydroindeno[1,2-c]pyrazole* (**6a**). A solution of the amide **11 (**0.14 g, 0.37 mmol**) **in THF (2 mL) was added dropwise to a suspension of LiAlH_4_ (56.0 mg, 1.48 mmol) in THF (2 mL) at 0 °C. After stirring at room temperature for 12 h the mixture was diluted with Et_2_O (2,5 mL) and then NaOH (1 M, 0.1 mL) e H_2_O (0.3 mL) were added. The formed solid was filtered and diluted with CH_2_Cl_2_. The organic phase was dried over anhydrous Na_2_SO_4_, filtered and the solvent was removed under reduced pressure. The residue was purified by flash chromatography (CHCl_3_/acetone = 95:5) affording **6a**: yield 80%; yellow solid; Mp 146–148 °C; *R_f_ =* 0.25 (CHCl_3_/MeOH 95:5); ^1^H-NMR: 1.75–2.16 (m, 6H), 2.60–2.80 (m, 1H), 2.95 (m, 2H), 3.50 (m, 1H), 3.53 (s, 2H), 3.59 (s, 2H), 7.17–7.37 (m, 6H), 7.42 (s, 1H), 7.61 (d, 1H, *J* = 8.0 Hz), 9.25–10.35 (brs, 1H). ^13^C-NMR: δ 28.7 (CH_2_), 30.3 (2 × CH_2_), 31.0 (CH), 52.5 (2 × CH_2_), 62.3 (CH_2_), 119.7(C), 125.3 (CH), 126.2 (CH), 126.6 (CH), 127.6 (2 × CH), 128.8 (2 × CH), 130.8 (CH), 133.0 (C), 136.5 (C), 141.1 (C), 141.4 (C) 143.6 (CN) 149.7 (CN). Anal. Calcd for C_22_H_22_ClN_3_: C, 72.62; H, 6.09; N, 11.55. Found: C, 72.70; H, 6.15; N, 11.59. 

### 3.8. 1H-1-Oxa-2-aza-6-chloro-3-(1-phenethylpiperidin-4-yl)-1,4dihydroindeno[2,1-d]isoxazole **(7a)** and 2H-1-aza-2-oxa-6-chloro-3-(1-phenethylpiperidin-4-yl)-1,4dihydroindeno[1,2-c]isoxazole **(8a)**

To a solution of phenyl 1-(phenylethyl)piperidine-4-carboxylate **17** (0.50 g, 1.23 mmol) were added in sequence 5-chloro-2,3-dihydro-1*H*-inden-1-one **1a** (0.2 g, 1.23 mmol) and NaH (60% in oil, 0.12 g, 3.08 mmol). The resulting mixture was heated under reflux for 4 h. After cooling a 50% aqueous solution of acetic acid was added and the resulting mixture was concentrated under reduced pressure. To the residue was taken up in EtOH (5 mL), AcOH (0.21 mL, 3.69 mmol) and hydroxylamine hydrochloride (0.128 mg, 1.85 mmol) was added. The resulting mixture was heated under reflux for 8 h. The solvent was evaporated under reduced pressure and the residue was taken up in CH_2_Cl_2_. The organic phase was dried over Na_2_SO_4,_, filtered and the solvent removed under reduced pressure. The residue was purified by flash chromatography (CHCl_3_/acetone = 8:2) to give **7a** and **8a**.

*Compound* **7a**. Yield 12%; brown solid; Mp 149–151 °C; *R_f_ =* 0,20 (CHCl_3_/acetone = 8:2); ^1^H-NMR: δ 1.60–2.04 (m, 9H), 2.49 (t, 2H, *J* = 7.2 Hz), 2.62 (t, 2H, *J* = 7.2 Hz), 3.55 (s, 2H) 7.23–7.42 (m, 8H). ^13^C-NMR: δ 28.8 (CH_2_), 30.6 (CH_2_), 31.1 (2 × CH_2_), 31.7 (CH), 52.5 (2 × CH_2_), 62.4 (CH_2_), 119.7 (C), 125.4 (CH), 126.2 (CH), 126.7 (CH), 127.8 (2 × CH), 128.8 (2 × CH), 130.9 (CH), 134.0 (C), 136.0 (C), 142.5 (C), 153.7 (C), 161.0 (CN), 166.4 (CO). Anal. Calcd for C_23_H_23_ClN_2_O: C, 72.91; H, 6.12; N, 7.39. Found: C, 73.12; H, 6.10; N, 7.43. 

*Compound* **8a**. Yield 41%; brown solid; Mp 157–160 °C; *R_f_ =* 0.41 (CHCl_3_/MeOH 8:2); ^1^H-NMR (DMSO-d_6_): δ 1.82–2.10 (m, 5H), 2.11–2.33 (m, 4H), 2.60–2.70 (m, 2H), 2.73–2.88 (m, 2H), 3.57 (s, 2H), 7.12–7.28 (m, 8H). ^13^C-NMR: δ 28.8 (CH_2_), 30.5 (CH_2_), 31.2 (2 x CH_2_), 31.4 (CH), 53.2 (2 × CH_2_), 62.3 (CH_2_), 119.6 (C), 125.3 (CH), 126.5 (CH), 126.6 (CH), 127.8 (2 × CH_2_), 128.8 (2 × CH_2_), 130.9 (CH) 134.1 (C), 136.5 (C), 142.4 (C), 151.9 (C), 162.3 (CN) 169.2 (CO). Anal. Calcd for C_23_H_23_ClN_2_O: C, 72.91; H, 6.12; N, 7.39. Found: C, 73.10; H, 6.14; N, 7.46. 

### 3.9. Phenyl 1-benzoylpiperidine-4-carboxylate **(13)** and phenyl 1-phenethylpiperidine-4-carboxylate **(17)**

A mixture of the acid **18** or **19** (2.19 mmol) in CH_2_Cl_2_ (40 ml), 1-3-dimethylaminopropyl)-3-ethylcarbodiimide hydrochloride (0.84 g, 4.38 mmol), dimethylaminopiridine (0.54 g, 4.38 mmol) and phenol (0.62 g, 6.57 mmol) was heated under reflux for 14 h. After cooling, the reaction mixture was diluted with CH_2_Cl_2_ and washed with a saturated NH_4_Cl solution (3 × 20 mL). The separated organic phase was dried over anhydrous Na_2_SO_4_. The solvent was removed under reduced pressure and the residue was purified by flash chromatography affording the product **13** or **17**.

*Compound* **13**. purified by flash chromatography by using as the eluent petroleum ether/EtOAc = 1:1); yield 62%; yellow oil; *R_f_ =* 0.46 (petroleum ether/EtOAc = 1:1); ^1^H-NMR: δ 1.65–2.10 (m, 6H), 2.70–2.90 (m, 1H), 3.10–3.25 (m, 2H), 7.00–7.45 (m, 10H). ^13^C-NMR: δ 28.2 (2 × CH_2_), 41.1 (CH), 46.8 (2 × CH_2_), 121.3 (2 × CH), 125.9 (CH), 126.8 (2 × CH), 128.4 (2 × CH), 129.4 (2 × CH), 129.6 (CH), 135.8 (C), 150.4 (C), 170.4 (CO), 172.6 (CO). IR: (nujol) ν 1,752 (CO), 1,628 (CO) cm^−^^1^. Anal. Calcd for C_19_H_19_NO_3_: C, 73.77; H, 6.19; N, 4.53. Found: C, 73.85; H, 6.25; N, 4.46.

*Compound* **17**. purified by flash chromatography by using as the eluent CHCl_3_/MeOH = 9:1; yield 64%; yellow oil; *R_f_ =* 0,27 (CHCl_3_/MeOH = 9:1); ^1^H-NMR: δ 1.80–2.20 (m, 4H), 2.56–2.65 (m, 2H), 2.80–2.90 (m, 3H), 3.00–3.15 (m, 2H), 3.43 (t, 2H, *J* = 5.4 Hz), 7.06 (d, 2H, *J* = 8.4 Hz), 7.20-7.42 (m, 8H). ^13^C-NMR: δ 28.2, (2 x CH_2_) 33.8 (CH_2_), 41.1 (CH), 46.8 (2 x CH_2_), 57.4 (CH_2_), 122.5 (2 × CH), 126.9 (2 × CH), 127.3 (CH), 128.4 (2 × CH), 129.0 (2 × CH), 129.5 (CH), 135.9 (C), 151.4 (C), 170.4 (CO). IR: (nujol) ν 1,750 (CO) cm^−^^1^. Anal. Calcd for C_20_H_23_NO_2_: C, 77.64; H, 7.49; N, 4.53. Found: C, 77.75; H, 7.42; N, 4.58**.**

## 4. Conclusion

In conclusion, we have reported a practical synthesis of the tricyclic heterocycles **E ** incorporating the pyrazole or isoxazole framework (Figure 1). These new products share with the antipsychotic compounds **A**–**C** two substituents, namely the chlorine on the aryl ring and the 4-(1-benzyl)- or 4-(1-phenylethyl)piperidinyl group on the isoxazole and pyrazole moieties. The antipsychotic activity of these new compounds will be determined, thus indicating which further structural modifications should be pursued to advantageously modify their biological activity.

## References

[1] Ye N., Neumeyer J.L., Baldessarini R.J., Zhen X., Zhang A. (2013). Upload: Recent progress in development of dopamine receptor subtype-selective agents: Potential therapeutics for neurological and psychiatric disorders. Chem. Rev..

[2] Rowley M., Collins I., Broughton H.B., Davery W.B., Baker R., Emms F., Marwood R., Patel S., Ragan I.C., Freedman S.B., Ball R., Leeson P.D. (1997). 4-Heterocyclylpiperidines as selective high-affinity ligands at the human dopamine D4 receptor. J. Med. Chem..

[3] Pinna G., Pinna G.A., Chelucci G., Baldino S. (2012). Tricyclic pyrazoles: An efficient approach to cannabinoid analogues with a tricyclic framework incorporating the pyrrole and pyrazole moieties. Synthesis.

[4] Silverman R.R. (1992). The Organic Chemistry of Drug Design and Drug Action.

[5] Wermuth C.G. (1996). The Practice of Medicinal Chemistry.

[6] Owton W.M., Brunaus M. (1991). Synthesis of 6/7 Halotetralones. Synth. Commun..

[7] Murineddu G., Ruiu S., Loriga G., Manca I., Lazzari P., Reali R., Pani L., Toma L., Pinna G.A. (2005). Tricyclic pyrazoles. 3. Synthesis, biological evaluation, and molecular modeling of analogues of the cannabinoid antagonist 8-chloro-1-(2,4-dichlorophenyl)-N-piperidin-1-yl-1,4,5,6-tetrahydrobenzo[6,7]cyclohepta[1,2-*c*]pyrazole-3-carboxamide. J. Med. Chem..

[8] Cossy J., Belotti D., Bouzide A., Thellend A. (1994). Short and efficient access to beta-ketoamides. Bull. Soc. Chim. Fr..

[B9-molecules-18-08147] 9.The structures of regioisomeric isoxazoles **7a,b,c** and **8a,b,c** have been confidently assigned by comparing their ^13^C-NMR spectra with those of the related and known open-chain isoxazoles **B** and **C** (Figure 1). Thus for istance, the ^13^C signals of the carbon bonded to oxygen in **B** and **C** are 166.6 ppm and 171.0 ppm, respectively, while those of **7c** and **8c** are 166.3 ppm and 170.7 ppm.

[10] Shao D., Zou C., Cheng L., Tang X., Li Y. (2004). Synthesis and evaluation of tacrine-E2020 hybrids as acetylcholinesterase inhibitors for the treatment of Alzheimer's disease. Bioorg. Med. Chem. Lett..

[11] Dutta A.K., Fei X.S., Beardsley P.M., Newman J.L., Reith M.E.A. (2001). Structure-activity relationship studies of 4-[2-(diphenylmethoxy)ethyl]-1-benzylpiperidine derivatives and their N-analogues: Evaluation of behavioral activity of O- and N-analogues and their binding to monoamine transporters. J. Med. Chem..

